# The knowledge of movement experts about stretching effects: Does the science reach practice?

**DOI:** 10.1371/journal.pone.0295571

**Published:** 2024-01-26

**Authors:** Konstantin Warneke, Andreas Konrad, Jan Wilke

**Affiliations:** 1 Institute of Sport Science, Alpen-Adria-University Klagenfurt, Klagenfurt am Wörthersee, Austria; 2 Institute of Human Movement Science, Sport and Health, University of Graz, Graz, Austria; Professionshøjskolen UCN: Professionshojskolen UCN, DENMARK

## Abstract

**Objective:**

Stretching is performed with numerous purposes in multiple settings such as prevention, rehabilitation, fitness training and sports. Its patterns of use substantially depend on the education and beliefs of health care and exercise professionals as they represent the multiplicators recommending and prescribing interventions to clients, patients and athletes. This study investigated movement experts’ knowledge about the scientific evidence on stretching effects.

**Design:**

Survey study.

**Participants:**

A total of 117 exercise and health professionals (physiotherapists, sports scientists, coaches) attending a training convention in Austria (male: n = 44, female: n = 73, 36±11 years) completed a digital survey. With its 22 items, the questionnaire addressed the movement experts’ awareness of the evidence on stretching effects regarding a variety of related topics selected based on the findings of topical systematic reviews.

**Results:**

The majority of the individuals (57–88%) assumed positive effects of stretching on recovery, prevention of muscle injury, range of motion, muscular imbalance and artery elasticity. No or adverse effects were mostly claimed on bone injury prevention, maximal/explosive strength, and delayed-onset muscle soreness. In only 10 of 22 items, participants’ classifications were in accord with the scientific evidence.

**Conclusions:**

The awareness of research findings on stretching effects among exercise and health professionals is alarmingly low. Future studies may hence be geared to improve implementation and science communication.

## Introduction

Stretching is primarily used to increase flexibility [1]. Although the exact mechanisms have been a matter of debate [2–6], a large body of evidence identified beneficial acute [7–11] and chronic [12–15] effects of stretch training on range of motion. Additional applications, inter alia, include treatments of muscle stiffness, pain or orthopedic complaints [16, 17], as well as the prevention of sports injuries [18, 19].

Contrarily to popular beliefs and despite its widespread application, stretching does not always achieve the assumed benefits. As an example, static stretching, particularly when performed for more than 60 seconds [5, 9, 20, 21], has adverse effects on explosive or maximal strength capacity [5, 21–24]. Interestingly, dynamic stretching does not seem to cause such performance reductions [3]. Injury prevention represents another area of application with ambivalent results [9, 25]. Most of the literature reported no preventive effects when considering total injury counts or other injury types such as ligament or bone injuries [26–29]. However, while Witvrouw et al. [29] indicated stretching could even increase the injury incidence in sports heavily relying on rapid stretch-shortening cycles (sprinting, change of directions), a more recent study by Behm et al. [9] reported static stretch to reduce incidence of musculotendinous injury incidence during activities and sports involving explosive contractions. Stretching is also often used for recovery, e.g., in delayed onset muscle soreness (DOMS) [30, 31]. However, even though some individual studies indicated potential positive (chronic) effects [32, 33], review articles failed to clearly identify significant improvements in this outcome [28, 31, 33–35]. Finally, for treatment of muscle imbalance, it is a widely performed practice to strengthen the presumably weakened or lengthened muscles and to stretch the tight or shortened muscles, which is known as the Janda approach [36, 37]. While the combination of strengthening and stretching indeed shows significant improvements [38], there is no evidence that stretching alone improves posture.

Coaches, exercise scientists and physiotherapists are well-known actuators in the health care and sports system [39]. They count among the most important promoters of movement [40] and exercise routines, using stretching in different settings such as sports or therapy [41]. Since Soligard et al [42] underlined the paramount importance of client compliance for successful implementation and effectiveness of prevention programs [43], the knowledge of the educators and experts can be assumed to be critical for the output of treatments.

Previous research on methods and contents applied in sports and therapy revealed a lack of awareness about the underlying scientific evidence. For instance, survey research showed that the tests used to estimate injury risk in elite basketball lack empirical support [44] and that a significant portion of therapies applied in knee osteoarthritis are not efficacious [45]. With regard to stretching, earlier surveys already addressed the patterns of use in sports [46–48]. However, while Babault et al. [47] explored the motives of athletes and exercise professionals to use stretching (e.g. for recovery or warm-up), to the best of our knowledge, no study has yet explored the actual awareness of the scientific evidence. Against this background, our study aimed to investigate the knowledge of experts and health care professionals (i.e. sport scientists, coaches and physical therapists) regarding the evidence for or against stretching in different practice applications. Considering the widespread popularity and use of stretching techniques [47], we hypothesized that there would be a significant discrepancy between the available evidence and the reported knowledge of the surveyed movement experts.

## Methods

### Ethics and design

The present study had two parts. In a first step, the evidence on stretching applications (e.g., injury prevention, performance, recovery) was examined identifying and evaluating relevant systematic reviews with meta-analysis. Subsequently, for part two, a cross-sectional survey assessing knowledge of the evidence was performed among participants of the exercise and training convention taking place in Klagenfurt, Austria. The study was approved by the local ethics committee and all participants provided digital informed consent.

### Evidence synthesis (study part 1)

For *step one*, all three investigators performed a literature search identifying systematic reviews with or without meta-analysis on the acute and chronic effects of stretching. Exemplarily, we used the following search term in PubMed to identify the evidence for the acute effects of stretch training on flexibility:

Stretch* AND acute AND (flexibility OR ROM OR “range of motion”) AND (“systematic review” OR “Meta analysis”)

As indicated, searches were performed for both acute and chronic effects by adding respective key words (acute OR immediate OR short-term / chronic OR long-term). In addition to the PubMed search, a free-hand search was performed using Google Scholar and the reference lists of all included studies were screened for eligible studies.

Based on the results of the identified reviews, findings on the effects of stretching were classified as negative effect, no effect, or positive effect. Classifications were based on results of meta-analyses (significance of pooled summary effect size) or the conclusions of the systematic reviews.

### Participants (study part 2)

All 191 healthy adults attending the training convention on 15^th^ of April, were invited to participate in our digital survey. Following a verbal and written information, informed consent was provided by clicking on a button labeled “I want to participate” below the digital study information sheet.

We obtained valid responses from n = 117 individuals (male: n = 44, female: n = 73, 36±11 years) which corresponds to a response rate of 61.26%. The majority of the participants were physiotherapists (n = 79), 10 of which stated a double education (both, Physiotherapy and Sport science). The remaining participants were sports scientists (at least, with a finalized three-years Bachelor degree) (n = 42) and coaches without the two beforementioned educations (n = 7). Most of the included individuals (n = 79) stated working with patients, while seven had elite athletes as clients. The remaining respondents reported working in the field of recreational sports.

### Questionnaire (study part 2)

The online questionnaire assessing the health experts`knowledge of the stretch evidence was generated using a consensus process among the authors and outcomes were based on the literature searches of step one. For face validation, the questionnaire was sent to three members of the target population (physiotherapists, fitness coaches, sports scientists) who were asked to independently judge the comprehensibility of the items. Their feedback was used to modify and refine the questionnaire. The final instrument mainly consisted of two tables (one for static and one for dynamic stretching) listing the identified fields of application with three options (positive/no/negative effect) for each row. The topics identified were: recovery, DOMS, muscle injury ligament injury, bone injury, range of motion (acute and chronic), maximal- and explosive strength (acute and chronic), muscle imbalance, and arterial elasticity. In addition, the questionnaire examined the sources used by the respondents to stay up to date about methods and their use (e.g., book reading, internet research, congresses).

### Statistics

Descriptive statistics (absolute and relative frequencies) were used to analyze the survey data. To compare the participants’ responses on stretching effects against the available scientific evidence, we applied the χ^2^ proportions test. It was used to reveal significant differences between the proportions of participants choosing the correct answer (e.g., positive effect of stretching) and those selecting an incorrect answer (e.g., no effect and negative effect). Thus, we were able to determine, if the majority of participants was aware of the scientific evidence. P values < .05 were considered significant, all calculations were performed with Jamovi (The Jamovi project).

## Results

### Part one

The literature search returned a total of 27 systematic reviews (n = 14 with meta-analysis) on a total of 11 topics. Two reviews addressed acute [49, 50] and four [12, 14, 15, 51] chronic ROM effects. The same applied to strength outcomes (acute: [21, 49], chronic: [52–55]. Nine papers [9, 26–28, 34, 35, 56–58] examined prevention of injury, while three reviews investigated a potential prophylactic impact on muscle soreness [28, 57, 59]. We additionally identified two articles each on recovery following sports and exercise [31, 60] and [61, 62]. One review [38] explored stretching effects on muscular imbalance. The accumulated evidence found by the included reviews was used to classify the stated answers of the health care professionals in Table 1, with green color indicating the response to be in accordance with the literature (correct) and red indicating the answer not to be in accordance with the literature (incorrect).

**Table 1:** Effects of static and dynamic stretching as assumed by the sample. Green (correct) and red (incorrect) colors indicate the correctness of the response options based on the literature search.

Topic		Total sample (n = 117)				Physiotherapists (n = 79)				Sport scientists (n = 42)			
		Negative Effect (percentage (n))	No influence (percentage (n))	Positive Effect (percentage (n))	χ^2^ test (p-value)	Negative Effect (percentage (n))	No influence (percentage (n))	Positive Effect (percentage (n))	χ^2^ test (p-value)	Negative Effect (percentage (n))	No influence (percentage (n))	Positive Effect (percentage (n))	χ^2^ test (p-value)
Static stretching	range of motion (acute)	4.3 (5)	20.5(24)	75.2 (88)	<0.001	5.8 (2)	22.8 (17)	73.4 (58)	<0.001	4.8 (2)	19.0 (8)	76.2 (32)	<0.001
	range of motion (chronic)	0.9 (1)	20.5 (24)	78.6 (92)	<0.001	2.7 (1)	22.8 (1)	75.9 (60)	<0.001	2.4 (1)	26.2 (11)	71.4 (30)	0.005
	maximal / explosive strength (acute)	51.3 (60)	32.5 (38)	16.2 (19)	<0.001	50.6 (40)	34.2 (27)	15.2 (12)	<0.001	61.7 (26)	21.4 (9)	16.7 (7)	<0.001
	maximal / explosive strength (chronic)	26.5 (31)	54.7 (64)	18.8 (22)	<0.001	27.8 (22)	57.0 (45)	13.9 (11)	<0.001	31.0 (13)	50 (21)	19.0 (8)	<0.001
	muscle injury	8.5 (10)	34.2 (40)	57.23 (67)	<0.001	10.1 (8)	32.9 (26)	54.4 (43)	0.004	9.5 (4)	33.3 (14)	57.1 (24)	0.031
	ligament injury	9.4 (11)	41.2 (48)	49.6 (58)	0.052	11.4 (9)	43.0 (34)	43.0 (34)	0.305	7.1 (3)	47.6 (20)	45.2 (19)	0.76
	bone injury	1.7 (2)	65.8 (77)	32.5 (38)	<0.001	1.3 (1)	65.8 (53)	31.6 (25)	0.003	2.4 (1)	71.4 (30)	26.2 (11)	0.005
	recovery	12.0 (14)	24.8 (29)	63.2 (74)	<0.001	13.9 (11)	26.6 (21)	59.5 (47)	<0.001	9.5 (4)	26.2 (11)	64.3 (27)	0.002
	delayed onset muscle soreness	28.2 (33)	43.6 (51)	28.2 (33)	0.166	26.6 (21)	46.8 (37)	25.3 (20)	0.651	28.6 (12)	47.6 (20)	23.8 (10)	0.135
	muscular imbalances	0 (0)	40.2 (47)	59.8 (70)	0.033	0 (0)	39.2 (32)	59.5 (47)	0.07	0 (0)	45.2 (19)	54.8 (23)	0.537
	artery elasticity	2.6 (3)	34.2 (40)	63.2 (74)	0.004	2.5 (2)	35.4 (28)	62 (49)	0.033	4.8 (2)	31.0 (13)	64.3 (27)	0.064
Dynamic stretching	range of motion (acute)	2.6 (3)	9.4 (11)	88.01 (103)	<0.001	2.5 (2)	12.7 (10)	83.5 (66)	<0.001	2.4 (1)	4.8 (2)	92.9 (39)	<0.001
	range of motion (chronic)	0 (0)	26.45 (31)	73.5 (86)	<0.001	0 (0)	30.4 (24)	69.7 (55)	<0.001	0 (0)	33.3 (14)	66.7 (28)	0.031
	Maximal / explosive strength (acute)	6.0 (7)	29.1 (34)	65.0 (76)	<0.001	6.3 (5)	27.8 (22)	65.8 (52)	<0.001	9.5 (4)	26.2 (11)	64.3 (27)	<0.001
	Maximal / explosive strength (chronic)	3.4 (4)	37.6 (44)	59.0 (69)	<0.001	3.8 (3)	38.0 (30)	58.2 (46)	<0.001	2.4 (1)	40.5 (17)	57.1 (24)	<0.001
	muscle injury	23.1 (27)	17.9 (21)	59.0 (69)	<0.001	19.0 (15)	17.7 (14)	60.8 (48)	<0.001	26.2 (11)	21.4 (9)	52.4 (22)	0.879
	ligament injury	20.5 (24)	30.78 (36)	48.7 (57)	<0.001	17.8 (14)	38.0 (30)	44.3 (35)	0.033	26.2 (11)	23.9 (10)	50 (21)	<0.001
	bone injury	9.4 (11)	59.8 (70)	30.8 (36)	0.033	7.6 (6)	63.3 (50)	29.1 (23)	0.018	14.3 (6)	57.1 (24)	28.6 (12)	0.355
	recovery	6.8 (8)	23.9 (28)	69.2 (81)	<0.001	6.3 (5)	25.3 (20)	67.1 (53)	<0.001	9.5 (4)	26.2 (11)	64.3 (27)	0.002
	delayed onset muscle soreness	17.1 (20)	26.45 (31)	56.4 (66)	0.013	12.7 (10)	26.6 (21)	59.5 (47)	<0.001	23.8 (10)	31.0 (13)	45.2 (19)	0.014
	muscular imbalances	0.9 (1)	38.45 (45)	60.67 (71)	0.021	0 (0)	41.8 (33)	57.0 (45)	0.174	2.4 (1)	38.1 (16)	59.5 (25)	0.123
	artery elasticity	0 (0)	26.5 (31)	73.5 (86)	<0.001	0 (0)	30.4 (24)	68.4 (54)	<0.001	0 (0)	23.8 (10)	76.2 (32)	<0.001

### Part two

#### Sources of self-education

One hundred-eight participants (92.3%) stated informing themselves by visiting congresses, while consulting colleagues (n = 66, 56.4%), book reading (n = 76, 65.0%), social media use (n = 63, 53.9%), scientific databases screening (n = 64, 54.7%) and website/blog reading (n = 54, 46.2%) were less frequently cited.

#### Descriptive results: Static stretching

The majority (75.2% and 78.6%)of the participants stated static stretching to positively affect range of motion in the short- and long-term (**Table 1**). Similarly, a high percentage (70–74%) assumed a beneficial impact on recovery, arterial elasticity and muscle imbalance. About the half of participants(57.2%/49.6%) suggested a protective effect of static stretching on muscle injuries and ligament injuries. No influence in either direction was mostly assumed for bone injuries and DOMS. The majority (83.8% and 81.2%) of the participants suggested negative or no acute or chronic effects of static stretching on maximal strength and explosive strength.

#### Descriptive results: Dynamic stretching

Similar to static stretching, the vast majority of the participants (75.5 and 88.01%) assumed acute and chronic range of motion increases following dynamic stretching. Regarding maximal strength capacity adaptations, about 60–65% assumed positive chronic effects. While three quarters (73.5%) of the movement experts claimed a positive influence of dynamic stretching on artery stiffness, two to three quarters (56.4 and 69.2%) suggested a positive influence on recovery and DOMS. Regarding injury prevention, 60% stated no effects of dynamic stretching on bone injury, while about five and six out of ten (48.7% and 59%) assumed positive effects on muscle and ligament injuries, respectively.

#### Statistical evaluation of evidence awareness

In 12 of the 22 items (static stretching: ROM acute/chronic, strength acute, prevention of muscle injury and bone injury, artery elasticity; dynamic stretching: ROM acute/chronic, strength acute/chronic, prevention of muscle injury, artery elasticity), the majority of the health professionals chose the correct answer (p < .05, **Table 1**). In 8 items (static stretching: strength chronic, recovery, muscular imbalance; dynamic stretching: prevention of ligament and bone injury, recovery, muscular imbalance, DOMS), the majority did not select the correct option (p < .05). In the remaining two items (static stretching in DOMS and for prevention of ligament injury), no majority could be identified by the χ^2^ proportion test (p>.05), meaning that–similar to the 8 aforementioned items–the correct answer could not be identified by a majority of participants. Separate analyses for sports scientists and physiotherapists only are also shown in **Table 1**. No differences (correct/incorrect answer) in evidence knowledge were found between members of the two professions (p>0.5).

## Discussion

Stretching represents one of the most researched topics in sports and exercise and is addressed in numerous review articles [12, 14, 21, 63, 64]. Despite some knowledge gaps (e.g., with regard to the physiological mechanisms), a plethora of studies have unveiled the effectiveness of stretching for various fields of application e.g. flexibility [12, 14, 15], performance [52, 53, 55], cardiovascular health [62], and muscular imbalance [38]. Our study found that exercise professionals are highly confident about the effects of stretching as they assumed a beneficial effect in 16 of 22 fields of application. This high confidence, however, does not reflect the available evidence: In 10 out of 22 questions asked in the present investigation, the response option chosen by the majority of the participants was incorrect. In sum, we therefore suggest that a significant number of scientific findings on stretching may not reach the movement experts working in clinical practice.

Previous stretching surveys provided valuable information regarding practical use of stretching in athletic populations and coaches [46–48]. However, no study had yet explored the evidence knowledge of movement experts. Notwithstanding, our finding of an unsatisfactory science to practice transfer with regard to stretching accords with a study by Zenko & Ekkekakis et al. [65] on general training prescription. The authors found only about 40% correct answers in a sample of 1,808 certified exercise professionals being surveyed about their knowledge about evidence-based training program design. An evidence-practice gap has also been reported by surveys on exercise during pregnancy (60–80% not aware of relevant guidelines; [66, 67]. In contrast, Bernhardsson et al. [68] observed a higher knowledge in Swedish physiotherapists. According to their results, 65 to 96% of the therapeutic advice provided by the included individuals (n = 271) were classified as being in line with the current evidence. Taken together, the findings of the present and the cited studies impressively demonstrate that scientific knowledge is transferred to practice to a highly variable degree.

It could be argued that awareness about scientific data is dependent on the novelty of research findings. The beneficial effects of stretching on flexibility have been reported using meta-analysis back in 2006 [69] and it is known since 2002 that stretching is not effective in DOMS [31]. The majority of our participants indeed correctly classified the rather old evidence with regard to both topics. Also, they failed to recognize the recently identified a) positive impact of chronic static stretching on strength parameters [55] and b) lack of an impact on posture [38]. While these results seem to support the assumption that evidence novelty represents a decisive predictor of awareness, the surveyed individuals correctly classified the newly discovered influence of stretching on arterial stiffness [62] but incorrectly assumed a protective effect of stretching on ligament injuries [26–28]. Therefore, we believe that not merely novelty of findings but also science communication may be paramount to secure translation to clinical practice.

Several authors have underscored the pivotal role of science communication and implementation research. As a consequence, researchers and journals have introduced a variety of strategies aiming to better disseminate study findings. Verhagen et al. [70] described the potential of social media, representing one of the most used platforms, to reach a large audience. Non-technical visualization, e.g., info graphics, sketch notes, video abstracts or video summaries, is another method to transmit complex findings [71]. Interestingly, while Wang et al. [72] suggested comics to be superior compared to info graphics regarding enjoyment and focus, Fontaine et al. [73] stated that high-quality studies investigating the effectiveness of such new science communication strategies are missing. Therefore, we argue that our results should represent a call to action for both practitioners to seek and use as well as for researchers to develop and apply science communication strategies.

### Limitations

Some methodological aspects merit consideration. A particular strength of our study was that we applied a census sampling methods inviting all participants of an exercise convention. With about 60%, the response rate was satisfactory. However, as our participants were registered for an educational event, we assume that they may have even be more interested in recent scientific knowledge than other non-registered colleagues. Furthermore, as the study focused on exercise professionals from Austria, Germany and Switzerland, caution is necessary when aiming to generalize the findings, as knowledge might differ between countries. Another issue relates to the evidence itself. In most cases, the classification of evidence as negative, neutral or positive was straightforward and unambiguous. However, in some areas of application, findings were more difficult to classify. For instance, static stretching has been demonstrated to reduce maximal/explosive strength. However, if stretch durations are very short, no impact of static stretching in either direction can be observed [5, 21]. Therefore, our questionnaire had some very few items with more than one correct option.

In literature, there is no clear definition of dynamic stretching, which could lead to some misunderstandings of the questions. Without scientifically clarify the dynamic stretching definition, this limitation cannot be counteract, yet.

Finally, since no option for undecided respondents was provided, the participants had to decide for one answer, which could impact the number of answers being classified as incorrect. However, since we wanted to check the knowledge of current evidence, participants who could have indicated that they do not know would also be considered incorrect.

## Conclusion

While there is a high confidence of physiotherapists and practitioners regarding the effects of stretching (positive impact assumed in 15 out of 22 items), the evidence does not fully support these positive assumptions. The findings of our study therefore represent a call to action for implementation and awareness research aiming to better communicate exercise-related research findings to coaches and therapists.

## Supporting information

S1 Checklist Human participants research checklist. (DOCX)

S1 File Survey. (DOCX)
