# Supplementary material for: The knowledge of movement experts about stretching effects: Does the science reach practice?
Source: PLoS One. 2024 Jan 26;19(1):e0295571. doi: 10.1371/journal.pone.0295571 (PMC10817148; doi:10.1371/journal.pone.0295571)
Supplement: S1 File — (DOCX) [file pone.0295571.s002.docx]

**Survey**

[How](https://survey.aau.at/index.php/admin/responses?1695282648345=&ajax=responses-grid&sa=browse&sort=479633X30411X422458SQ001.desc&surveyid=479633) does static stretching influence ….

Regeneration: ( ) positive ( ) no influence ( ) negative

Delayed onset muscle soreness: ( ) positive ( ) no influence ( ) negative

Prevention muscle injury ( ) positive ( ) no influence ( ) negative

Prevention ligament injury ( ) positive ( ) no influence ( ) negative

Prevention bone injury ( ) positive ( ) no influence ( ) negative

Flexibility (acute) ( ) positive ( ) no influence ( ) negative

Flexibility (chronic) ( ) positive ( ) no influence ( ) negative

Maximal- and explosive strength (acute): ( ) positive ( ) no influence ( ) negative

Maximal- and explosive strength (chronic): ( ) positive ( ) no influence ( ) negative

Muscular imbalance: ( ) positive ( ) no influence ( ) negative

Artery elasticity: ( ) positive ( ) no influence ( ) negative

[How](https://survey.aau.at/index.php/admin/responses?1695282648345=&ajax=responses-grid&sa=browse&sort=479633X30411X422458SQ001.desc&surveyid=479633) does dynamic stretching influence ….

Regeneration: ( ) positive ( ) no influence ( ) negative

Delayed onset muscle soreness: ( ) positive ( ) no influence ( ) negative

Prevention muscle injury ( ) positive ( ) no influence ( ) negative

Prevention ligament injury ( ) positive ( ) no influence ( ) negative

Prevention bone injury ( ) positive ( ) no influence ( ) negative

Flexibility (acute) ( ) positive ( ) no influence ( ) negative

Flexibility (chronic) ( ) positive ( ) no influence ( ) negative

Maximal- and explosive strength (acute): ( ) positive ( ) no influence ( ) negative

Maximal- and explosive strength (chronic): ( ) positive ( ) no influence ( ) negative

Muscular imbalance: ( ) positive ( ) no influence ( ) negative

Artery elasticity: ( ) positive ( ) no influence ( ) negative
